# Standardized endocystectomy technique for surgical treatment of uncomplicated hepatic cystic echinococcosis

**DOI:** 10.1371/journal.pntd.0007516

**Published:** 2019-06-21

**Authors:** Mohammed Al-Saeedi, Elias Khajeh, Katrin Hoffmann, Omid Ghamarnejad, Marija Stojkovic, Tim F. Weber, Mohammad Golriz, Oliver Strobel, Thomas Junghanss, Markus W. Büchler, Arianeb Mehrabi

**Affiliations:** 1 Department of General, Visceral, and Transplantation Surgery, University of Heidelberg, Heidelberg, Germany; 2 Section of Clinical Tropical Medicine, University Hospital Heidelberg, Heidelberg, Germany; 3 Department of Diagnostic and Interventional Radiology, University Hospital Heidelberg, Heidelberg, Germany; IRNASA, CSIC, SPAIN

## Abstract

**Background:**

Two surgical options are available for cystic echinococcosis (CE). The two principal approaches are radical (resection of the cyst) and conservative (evacuation of the cyst content and partial removal of the cyst capsule). Here, we describe a standardized endocystectomy technique for hepatic echinococcosis.

**Subjects and methods:**

Twenty-one patients (male/female: 4/3; median age: 28 years) with uncomplicated, isolated hepatic CE (cyst stages WHO CE1, 2, 3a, and 3b) that were treated with the standardized endocystectomy described in this paper. Before the operation and during the follow-up period (mean: 33.8 months, median: 24 months), patients underwent clinical and sonographical and/or magnetic resonance imaging assessment during regular visits managed by an interdisciplinary team.

**Results:**

Forty-seven cysts were treated with the standardized endocystectomy technique. The median number of cysts per patient was two (range: 1–8). Nine patients (43%) had a single cystic lesion. The median operation time was 165 minutes and the median intraoperative bleeding volume was 200 mL. The median hospital stay was nine days (range: 6–28 days). Morbidity (Clavien-Dindo III) occurred in four patients (19%). No mortality and no recurrence were found during the median follow-up time of 24 months.

**Conclusions:**

The standardized endocystectomy technique presented is a safe procedure with acceptable morbidity, no mortality, and without recurrences in our patient series. Important components of our CE management are interdisciplinary patient care, adequate diagnostic work-ups, and regular pre- and postoperative visits, including long-term follow-up for early and reliable capture of recurrences.

## Author summary

Cystic echinococcosis (CE) is a parasitic disease caused by ingestion of the larval stage of *Echinococcus granulosus*. The liver is the most commonly infected organ. There are currently four treatments for CE: surgery, percutaneous treatment, medical treatment (benzimidazoles), and watch-and-wait strategy. Treatment is decided based on the WHO cyst staging. The surgical techniques employed depend on the cyst location and related complications (e.g. cyst-biliary fistulas, rupture, and secondary bacterial infection). The two principal surgical approaches are radical (resection of the cyst) and conservative (evacuation of the cyst content and partial removal of the cyst capsule) surgeries. In this study, we presented a conservative surgical approach, a standardized endocystectomy technique, that is suitable for surgical residents. This standardized endocystectomy technique is a safe procedure with acceptable morbidity, no mortality, and without recurrences in our patient series. Important components of CE management are interdisciplinary patient care, adequate diagnostic work-ups, and regular pre- and postoperative visits, including long-term follow-up for early and reliable capture of recurrences.

## Introduction

Cystic echinococcosis (CE) is a parasitic disease caused by ingestion of the larval stage of *Echinococcus granulosus*. The liver is the most commonly infected organ (about 75% of cases), followed by the lungs and other organs. In most high-income countries, the incidence of CE is very low, diagnosed mainly in migrants originating from endemic regions. CE is largely asymptomatic, diagnosed incidentally or when complications precipitate, such as biliary obstruction due to cysto-biliary fistulas, spillage of cyst content into the biliary tree, and compression of vessels (bile ducts, portal or hepatic veins, and inferior caval vein in hepatic CE). Outside endemic regions, most health care professionals have limited experience with CE patients [1–4].

Medical treatment (benzimidazoles), percutaneous interventions, surgery, and the watch-and-wait strategy are the available treatment options and the treatment strategy is decided based on the WHO CE cyst classification [1, 3–11]. Without CE cyst staging, uncomplicated CE cysts are overtreated and inappropriate use of imaging modalities causes misclassification [12–14].

The recommendations of the Informal WHO Working Group Echinococcosis (WHO-IWGE) of 2010 remain largely valid for the surgical treatment of CE: “Surgery should be carefully evaluated against other options before choosing this treatment. It is the first choice for complicated cysts. In liver cysts, surgery is indicated for (a) removal of large CE2–CE3b cysts with multiple daughter vesicles, (b) single liver cysts, situated superficially, that may rupture spontaneously or as a result of trauma when percutaneous treatment (PT) options are not available, (c) infected cysts when PTs are not available, (d) cysts communicating with the biliary tree (as alternative to PT), and (e) cysts exerting pressure on adjacent vital organs.” [3] Recently, CE2 and CE3b were successfully treated percutaneously with a technique that awaits further consolidation in other CE treatment centers [7].

The surgical indications and approaches need to be further elaborated and defined for the various cyst presentations and health system settings. The two principal surgical approaches are radical and conservative. In radical surgery, including liver resection and pericystectomy, the entire unopened CE cyst including the host tissue-derived capsule is removed, which means loss of liver tissue. In conservative surgery, the content of the CE cyst is carefully evacuated and the host tissue-derived CE capsule is only partially removed, which spares liver tissue.

Endocystectomy is the most common conservative surgical approach, originally developed by Lindeman in 1871. Reported complication, mortality, and recurrence rates reflect varied performances across health care settings more than characterizing the technique as such. Endocystectomy has been modified multiple times over the years, including omentoplasty, protective procedures to avoid spillage of cyst content–during the most critical step when the cyst is opened and evacuated–and partial excision of the host-derived capsule.

We describe in detail a standardized endocystectomy technique for treating uncomplicated hepatic CE that is suitable for surgical residents.

## Materials and methods

**Fig 1:**
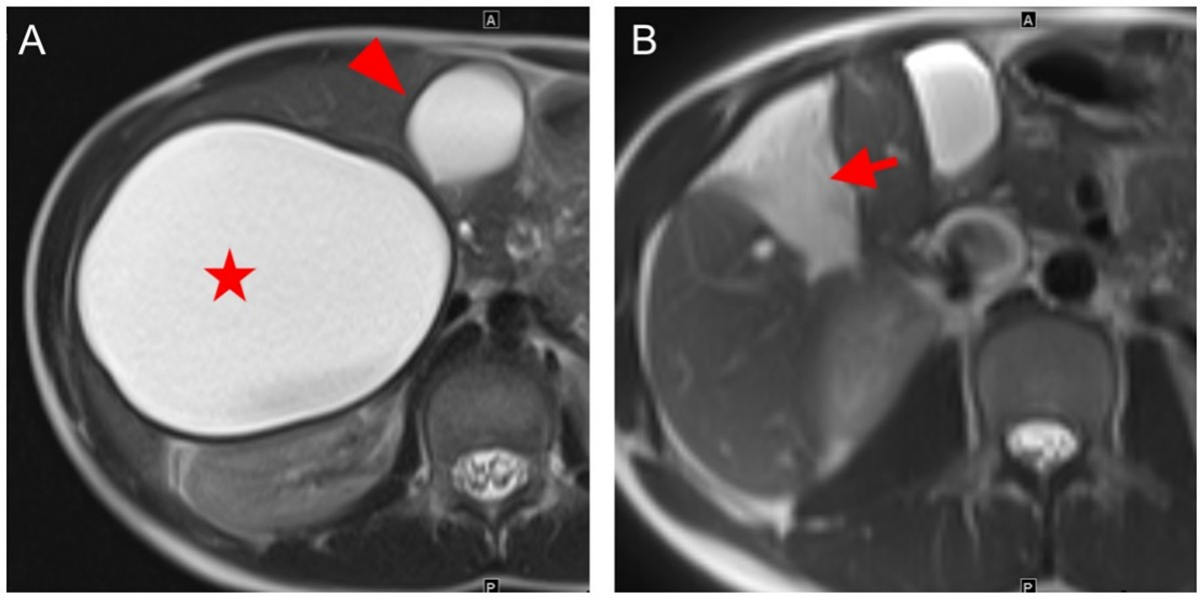
Patient with hepatic cystic echinococossis WHO stage CE1. A: The preoperative T2-weighted MRI scan shows a large mass in the right liver lobe that is homogeneously filled with fluid-equivalent content corresponding to a WHO CE1 cyst (star symbol). The arrow head points to the gall bladder. B: The postoperative T2-weighted MRI scan shows the omentoplasty within the residual endocystectomy cavity (arrow).

**Fig 2:**
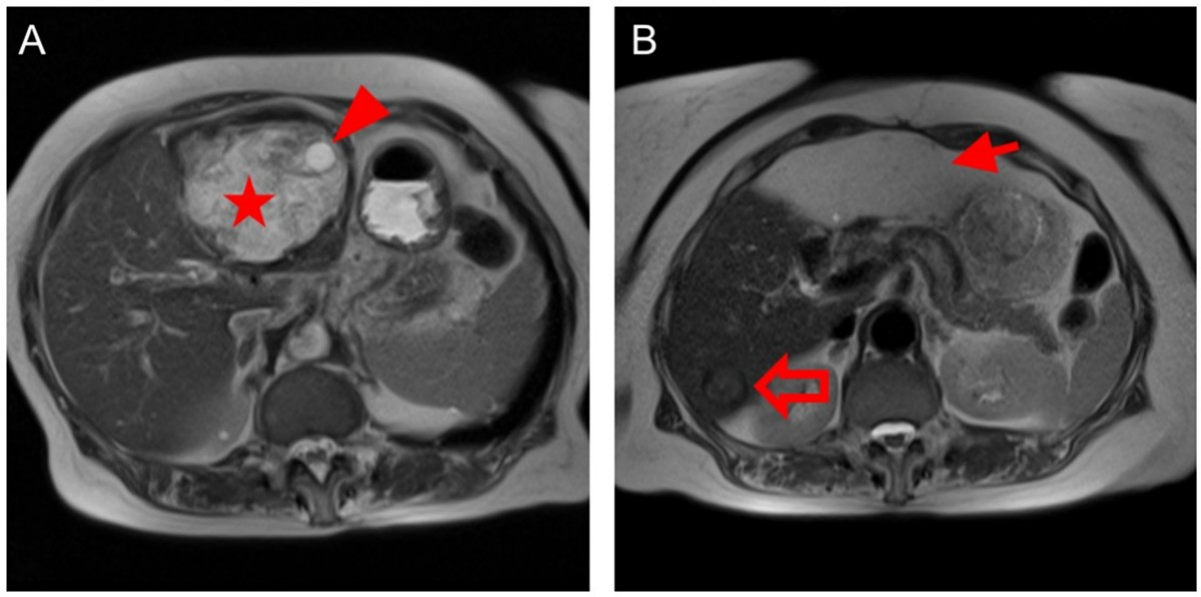
Patient with hepatic cystic echinococossis WHO stage CE3b. A: The preoperative T2-weighted MRI scan shows a large cystic mass in the left liver lobe that has a heterogeneous content including solid components (star symbol) and one daughter vesicle (arrow head) corresponding to a WHO CE3b cyst. B: The postoperative T2-weighted MRI scan shows the omentoplasty within the residual endocystectomy cavity (arrow). The open arrow points to an inactive WHO CE5 cyst in the right liver lobe.

The “Heidelberg Echinococcosis Treatment Center” is an interdisciplinary referral center that manages patients with cystic and alveolar echinococcosis since 1999. The center comprises the Clinical Tropical Medicine Unit, Department of Radiology, Department of General Surgery, Department of Thoracic Surgery, Interdisciplinary Center for Endoscopy, and the Department of Parasitology. Between June 2011 and January 2017, 21 patients with isolated hepatic CE were treated with the standardized endocystectomy technique described in this paper. The patients were non-acute patients with WHO cyst stages CE 1, 2, and 3a liver cysts of > 10 cm (Fig 1), CE 3b cysts largely independent of size (Fig 2), and CE1 to CE3b cysts that had unsuccessfully been treated by other modalities. Demographic and clinical characteristics as well as WHO CE cyst stages are presented in Table 1. In total, the patients had 47 cysts. In our setting, diagnosis, assessment for surgery, operation procedures, postoperative in-hospital course, and outpatient follow-up are monitored and documented by the interdisciplinary team, coordinated by the Clinical Tropical Medicine Unit. A physician from this unit is always present during the surgical interventions.

**Table 1:** Baseline characteristics and intra- and postoperative patient outcomes.

Patient no.	Gender	Age (years)	Country of origin	History of CE disease	No. of cysts	Median diameter (cm)	WHO cyst stage	CE related disease	Duration of operation (minutes)	CHE/White test	Omentoplasty	Blood loss (mL)	Hospital stay (days)	Complications	Follow-up (months)
1	M	16	Iraq	-	3	9.6	CE 1	-	130	-/-	Yes	100	10	-	72
2	M	38	Turkey	-	1	6	CE 2	-	150	Yes/Yes	Yes	400	7	-	75
3	M	31	Uzbekistan	Yes	3	6.8	CE1, CE4–5	-	180	Yes/Yes	-	200	7	-	56
4	F	20	Tajikistan	Yes	2	7.3	CE 1	-	165	-/-	Yes	200	8	-	72
5	F	55	Bulgaria	-	2	8.8	CE 1–2, CE 3a-4	-	225	Yes/-	Yes	400	12	-	59
6	F	76	Turkey	-	2	8	CE 3b, CE 5	Cholestasis, post-ERCP pancreatitis	180	Yes/-	Yes	400	27	-	48
7	F	34	Iraq	-	1	10	CE 1	-	150	Yes/-	Yes	200	8	-	46
8	F	24	Turkey	Yes	1	10	CE 3b	-	150	Yes/Yes	-	100	7	-	44
9	F	24	Bulgaria	-	1	8.3	CE 2	-	120	Yes/-	Yes	200	13	Bile leakage (IIIb)	36
10	F	17	Bulgaria	-	1	10	CE 3b	-	200	Yes/-	Yes	50	7	-	24
11	M	18	Afghanistan	Yes	1	7.5	CE 3a	Cysto-biliary fistula, choleastasis	180	-/-	Yes	800	8	-	15
12	M	34	Syria	-	5	-	CE 3b–4	-	210	Yes/-	-	400	10	-	30
13	M	33	Macedonia	-	3	5.2	CE 2, CE 3b, CE 5	-	150	-/-	-	100	13	Bile leakage (IIIb)	23
14	F	40	Macedonia	Yes	2	4.1	CE 2, CE 3b	-	120	Yes/Yes	Yes	50	28	Bile leakage (IIIa)	16
15	M	26	Afghanistan	-	1	15	CE 3b	-	220	Yes/Yes	-	500	7	-	1
16	M	28	Afghanistan	-	1	8	CE 2	-	165	Yes/Yes	Yes	50	8	-	19
17	M	27	Syria	-	2	8	CE 1	-	125	-/-	Yes	200	15	-	11
18	M	52	Azerbaijan	-	4	9.3	CE 2, CE 2, CE 3a	Cysto-biliary fistula, cholangitis	230	Yes/-	-	700	24	Pleural effusions, pneumonia (IIIa)	10
19	M	34	Romania	-	1	9.5	CE 3b	-	125	-/-	Yes	50	9	-	11
20	F	22	Iraq	-	2	-	CE 2	-	225	-/-	Yes	50	6	-	22
21	M	20	Afghanistan	Yes	8	-	CH 3a, CE 2, CL/CE 1, CE 2, CE 2, CE 4/CE 5, CE 3a, CE 3a	Pulmonary CE	250	-/-	-	250	9	-	20

CE, cystic echinococcosis; WHO, World Health Organization; CHE, cholecystectomy; ERCP, endoscopic retrograde cholangiopancreatography; M, male; F, female

### Ethics statement

All procedures were performed according to the most recent revision of the Declaration of Helsinki. All included patients aged more than 18 years old and a written informed consent for anonymous collection and analysis of clinical data was provided by all patients before surgery. The study protocol was also approved by the independent ethics committee of the university (S-754).

### Preoperative assessments

Preoperative diagnosis is based on imaging, preferentially ultrasound and/or magnetic resonance imaging (MRI) [3, 11, 14–16]. Serology is only used to confirm the diagnosis [17, 18]. All patients receive 400 mg albendazole 6 hours prior to surgery and this treatment is continued for one month after the operation if no intraoperative complications occur. Liver function tests and neutrophils are carefully monitored during benzimidazole treatment. All patients receive a preoperative X-ray of the chest to exclude concomitant lung CE.

### Surgical procedure

**Fig 3:**
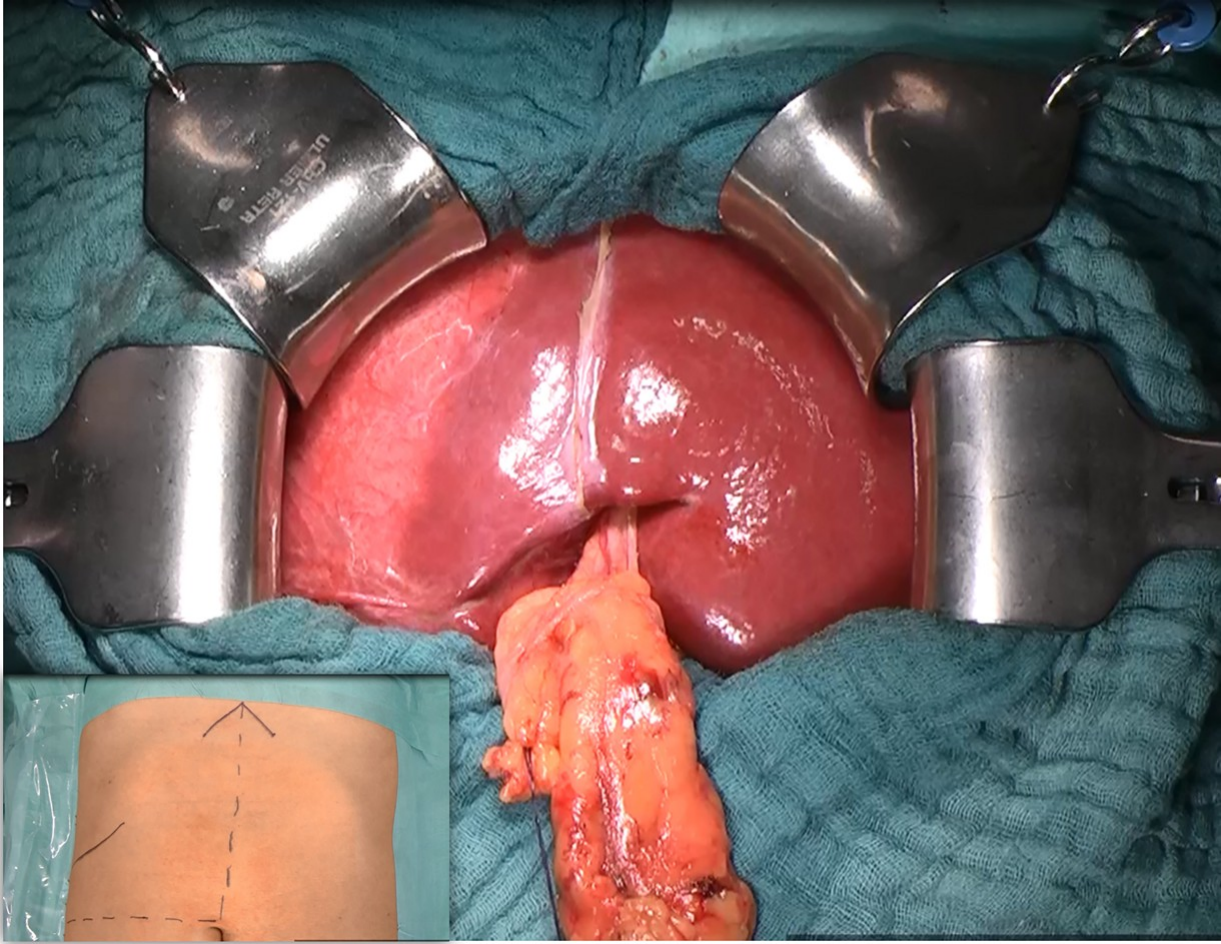
Laparotomy. Midline incision and dissection of the falciform ligament.

**Fig 4:**
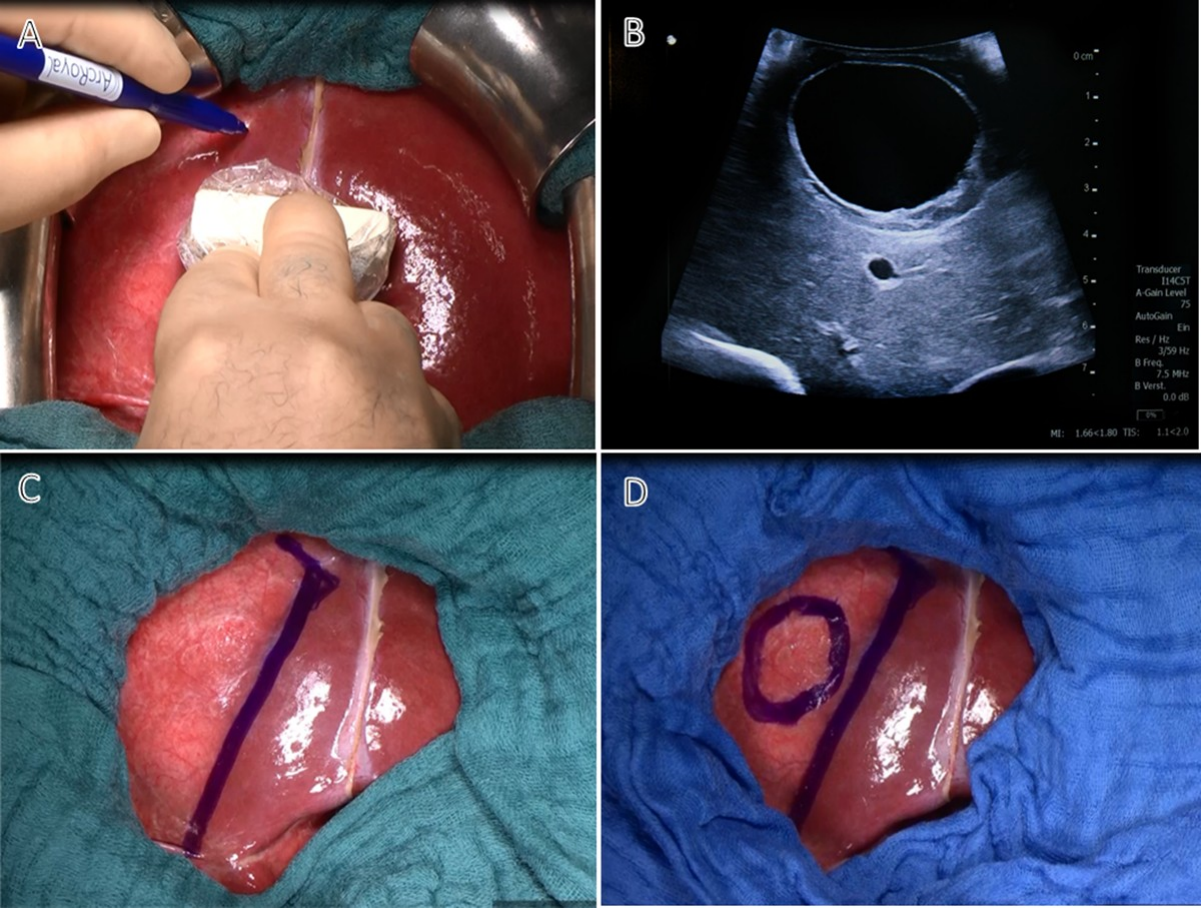
Preparation steps before cyst evacuation. A and B: Cyst localization using ultrasound. C: Protection of the surrounding tissue using sterile green laparotomy sponges (moistened with 0.9% normal saline). D: Second layer of blue laparotomy sponges soaked with 20% sodium chloride.

For endocystectomy, the patient is positioned in a supine position and laparotomy is performed using a supraumbilical midline incision with a right lateral extension if necessary. Afterwards, the liver is mobilized by dividing its ligaments (Fig 3). Intraoperative ultrasound examination is performed to confirm the number of cysts (Fig 4A and 4B), to define and mark the exact anatomical cyst locations (Fig 4C and 4D), and to finally check if all cysts are removed (particularly exophytic growth). If a cholecystectomy is performed, the cystic duct is temporarily secured using a bulldog clamp so that any bile leakage could later be determined by retrograde instillation of fat emulsion solution into the bile system (White test).

To protect the surrounding tissues from spillage of cyst content, the surgical field was covered with sterile green laparotomy sponges (moistened with 0.9% normal saline) (Fig 4C). Blue laparotomy sponges soaked with 20% sodium chloride were placed on top of the green sponges (Fig 4D). Different colors were used to safely distinguish the two types of surgical sponges. Twenty percent sodium chloride inactivates protoscolices and the germinal layer of the endocyst, but it is cytotoxic to the peritoneum and the biliary tract. Since contact with cyst content is unavoidable during the opening of the cyst and the evacuation procedure, the instrumentation table and neighbouring surfaces were covered and only the necessary surgical instruments, including the suction tip, were exposed. These were immediately removed after use. Gloves and gowns were changed before continuing with the “clean” procedures.

**Fig 5:**
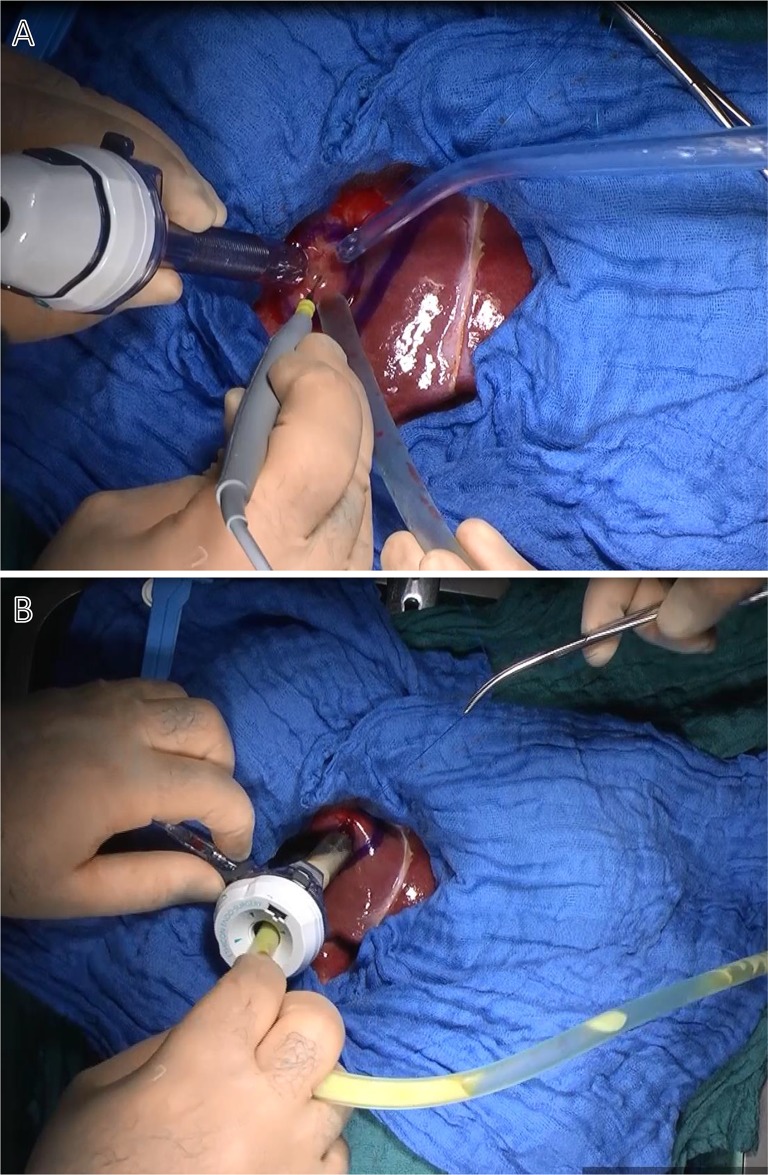
Evacuation of the cyst. A: Puncture of the cyst using a 12 mm trocar with permanent suction. B: Complete aspiration of the cyst content.

**Fig 6:**
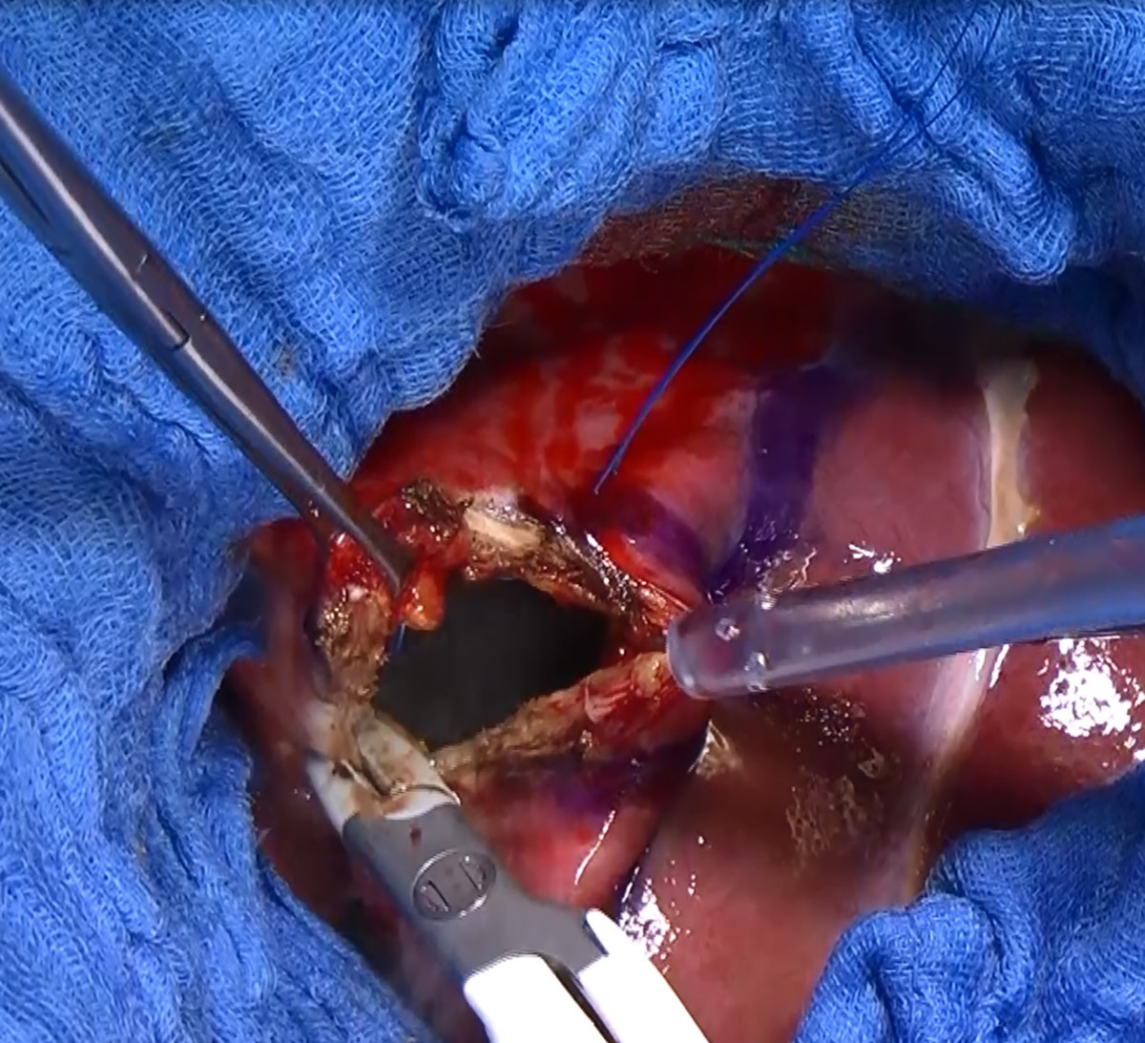
Fenestration of the cyst. Removal of the residual internal capsule of the cyst.

**Fig 7:**
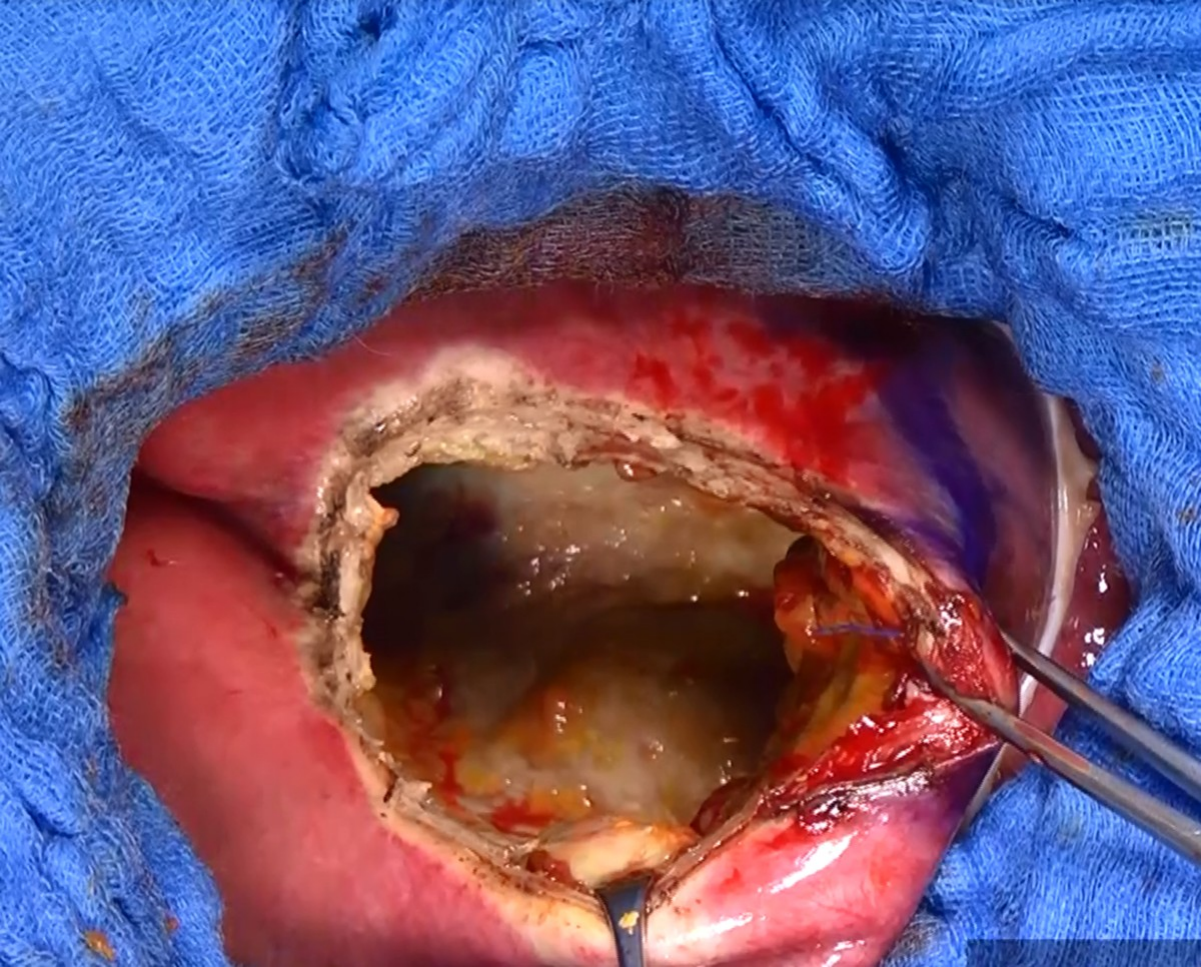
Exploration of the residual cavity. Closure of cysto-biliary fistula(s).

The insertion site for the 12 mm trocar was carefully selected at the part of the pericyst exposed to the liver surface (Fig 4D). The trocar was preferentially inserted vertically and tightened with a purse string suture to control spillage of CE fluid at the trocar-tissue interface (Fig 5A). Four holding threads prevent retraction of the pericyst during emptying of the cyst. Fig 5B shows removal of the cyst content, including the fragmented endocyst, protoscolices, cell detritus, and hydatid fluid using a 12 mm trocar under permanent suction. The evacuated fluid is centrifuged and protoscolices microscopically assessed for viability after eosin staining. The cyst is thoroughly washed with normal saline via a three-way valve. After the trocar is removed, the capsule is partially resected at the inner circumference of the holding threads (Fig 6). The inner surface of the cyst capsule is inspected and any residual endocyst material that may have remained after evacuation and washing of the cysts is removed (Fig 7).

**Fig 8:**
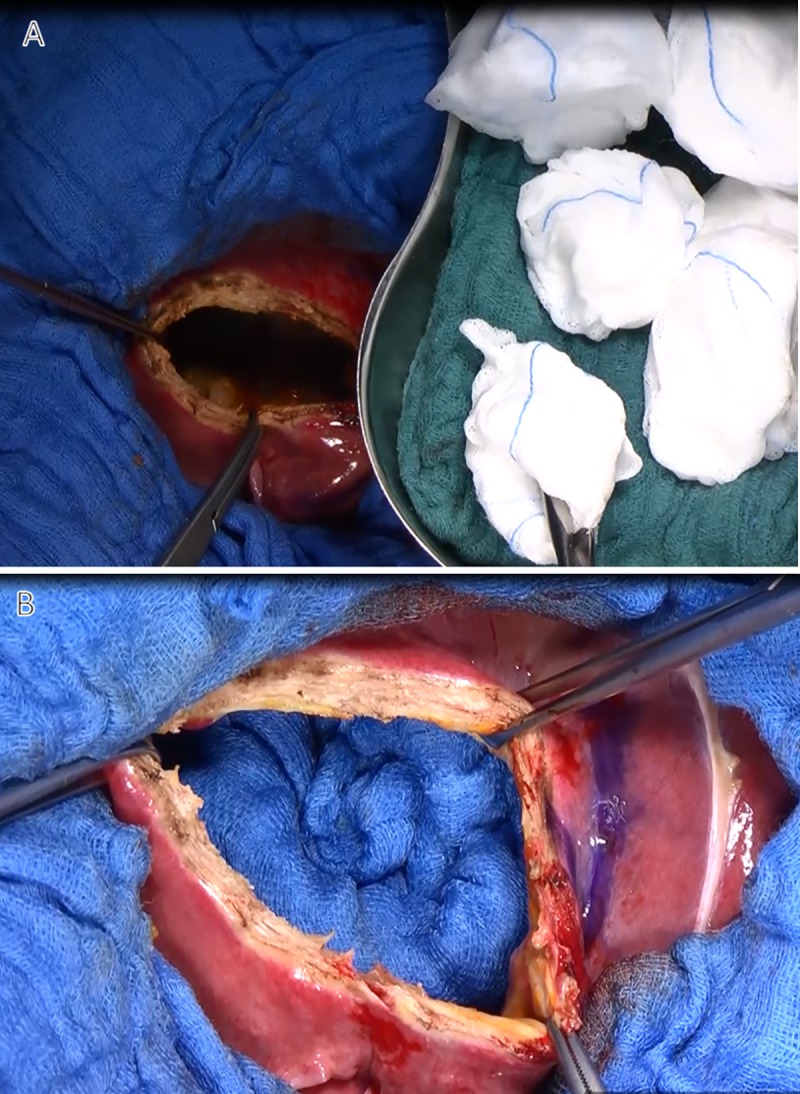
Sterilization of the cyst. The cyst is filled with swabs soaked in normal saline (A), and then with sponges soaked in 20% sodium chloride (B).

**Fig 9:**
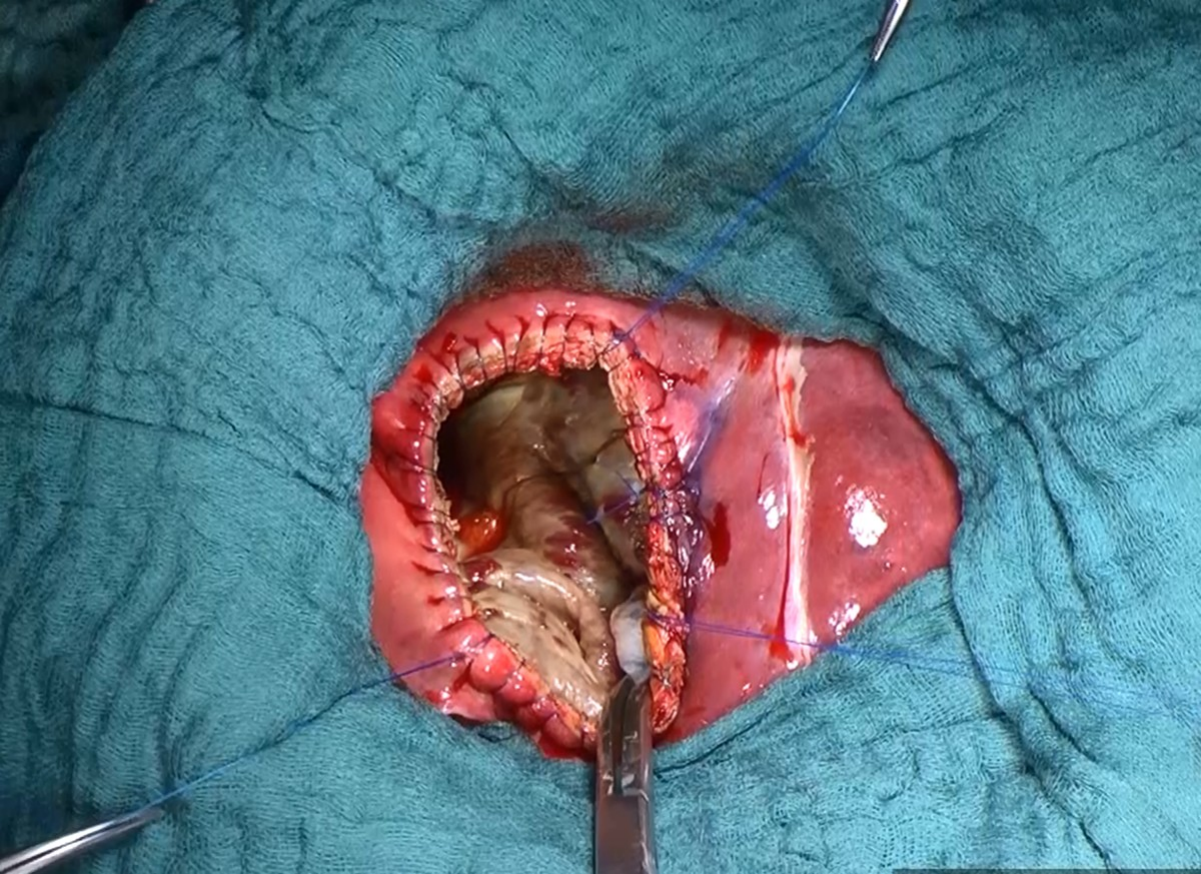
Over sewing the cyst edge.

**Fig 10:**
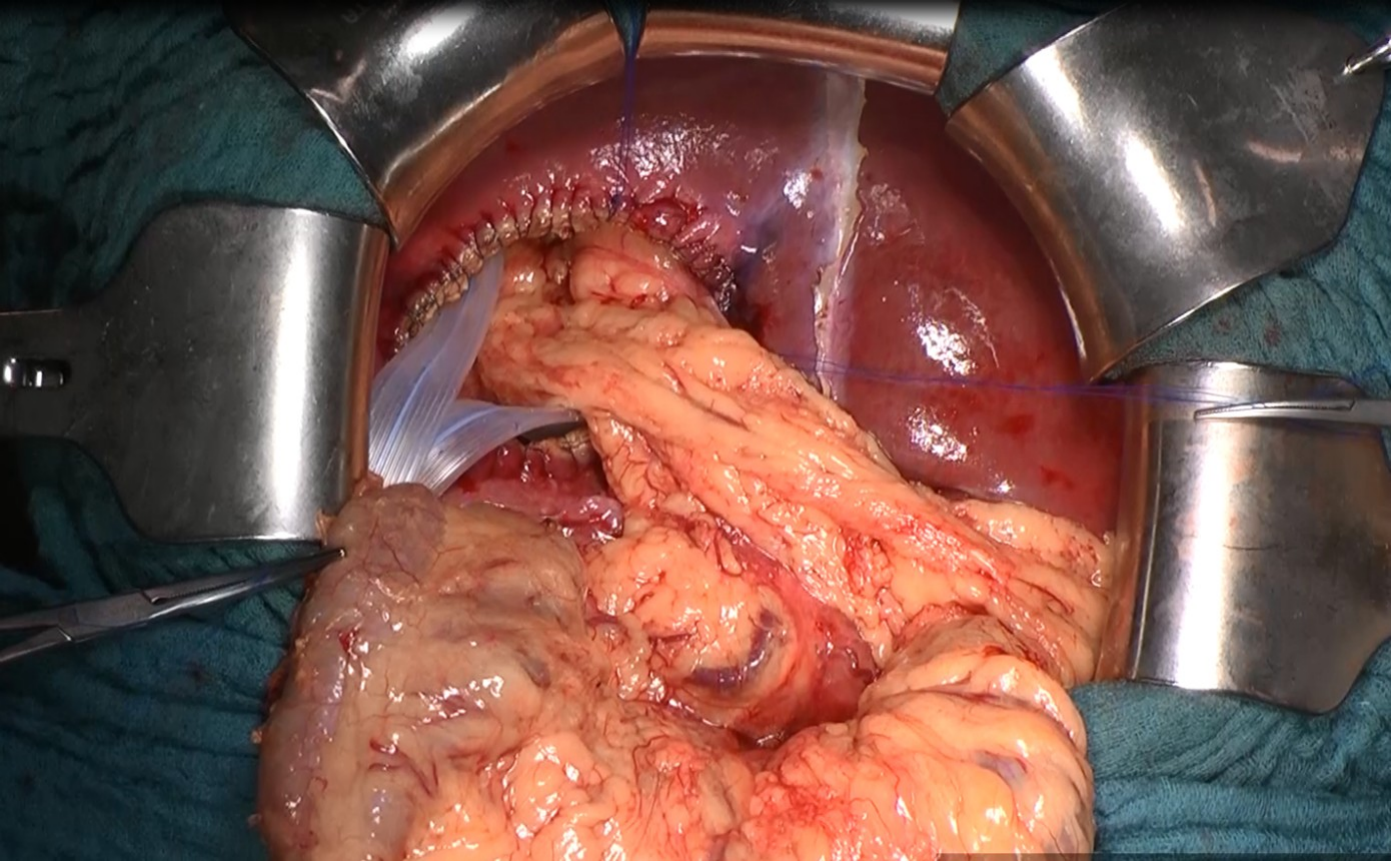
Omentoplasty and drainage.

The residual cavity is explored carefully for cysto-biliary communications (Fig 7). Cysto-biliary fistulas are apparent if the evacuated cyst content is bile stained. In a substantial proportion of cysts, cysto-biliary fistulas can be predicted with a preoperative MRI [15]. All visible cysto-biliary communications are closed with stitches. Afterwards, a White test is performed using 10–30 mL of liposaccharide to detect further bile leakage [19]. After the biliary fistulas are closed, the cyst cavity is thoroughly cleaned with swabs soaked in normal saline (Fig 8A), and then stuffed with sponges soaked in 20% sodium chloride for 30 minutes (Fig 8B). The same procedure is followed if no cysto-biliary fistulas are detected. After inactivation of any remaining parasitic cells (protoscolices and germinal membrane cells), the sponges are removed, and the edge of the cyst is over sewn to prevent bile leakage (Fig 9). In patients with multiple cysts, the procedure described above is repeated for each cyst. To take care of the residual cavity, if possible, an omentoplasty is performed by inserting a plug of the greater omentum fixed at the margin of the cyst wall (Fig 10). Before closing the abdominal wall, a drain is placed in the operation field.

### Postoperative assessments

Patients are regularly visited by surgeons and tropical medicine specialists during hospitalization. Intra- and postoperative complications are recorded and classified based on the Clavien-Dindo classification [20]. After discharge from the hospital, the patients are followed up in the outpatient clinic of the surgical department and the special clinic for echinococcosis in the tropical medicine unit. The latter takes care of perioperative albendazole treatment and long-term follow-up for relapses. To detect recurrences early–depending on the location of the cyst–ultrasound or MRI investigations are annually performed; the first set of images within one month of the operation to document fluid retention, which is not related to CE recurrence (biliomas, seromas). This allows us to distinguish CE-regrowth from preexisting non-CE fluid collections during later follow-up sessions. The whole abdomen, including the lesser pelvis, is observed to detect CE recurrence distant from the evacuated CE cysts. Follow-up is continued for at least 7 years to exclude late recurrences.

## Results

### Patient demographic and clinical data

All twenty-one patients originated from endemic regions in Eastern Europe, the Middle East, and Asia. The median patient age was 28 years (range: 16–76 years). Twelve patients (57.1%) were male, nine female (42.9%). Nine patients (42.9%) had a single cyst, the remaining 12 patients (57.1%) had multiple cysts. A total of 47 cysts were detected in the 21 patients included in the study. The median number of cysts was two (range: 1–8). One patient had eight cysts and sequential endocystectomies successfully removed all cysts in one session. The cyst diameters ranged between 4.1 and 15 cm. A total of 47 endocystectomies were performed with the standardized method described above. All patients received perioperative albendazole, except one pregnant patient. She was 22 years old and was referred with hepatic CE identified during a routine gynecologic examination in the 22nd week of pregnancy. The patient was operated because of the risk of cyst rupture during childbirth. The operation was performed successfully together with a gynecologist. Six of the treated patients (28.6%) were referred from other centers because of CE recurrence after initial treatment. Preoperative cysto-biliary fistulas were detected in two patients (9.5%), biliary obstruction was reported in two patients (9.5%), and cholangitis was diagnosed in one patient (4.8%).

### Intraoperative data

Concomitant cholecystectomy was performed in 13 patients (61.9%). In six of these patients, the White test was performed to exclude bile leakage. Omentoplasty was performed in 14 patients (66.7%). The median intraoperative blood loss was 200 mL (range: 50–800 mL) and the median operation time was 165 minutes (range: 120–250 minutes). No CE fluid spillage occurred during the operations.

### Postoperative outcome

The median hospitalization of the patients was 9 days (range: 6–28 days) and the median and mean follow-up period were 24 and 33.8 months, respectively (range: 1–75 months, first quartile: 15.5 months, third quartile: 52 months). Four complications (19.0%) occurred during the follow-up period, without any mortality. Three of these complications were surgical (bile leakage) and one was non-surgical (pleural effusions/pneumonia). All complications were Clavien-Dindo grade III; two cases required non-surgical intervention (IIIa) and two cases required reoperation, over sewing, and lavage because of bile leakage (IIIb). Relative to the total number of operated cysts, the rate of bile leakage as the only surgical complication was 6.4%. No recurrence was recorded during the study period.

## Discussion

Surgery is the method of choice for most complicated CE cysts (those with cyst-biliary fistulas, ruptured cysts) and for uncomplicated active cysts (mainly very large cysts > 10 cm) that cannot be treated percutaneously, with benzimidazoles, or observation (watch-and-wait strategy) [1, 3–10].

The criteria for assigning patients to radical (liver resection/pericystectomy) or conservative parenchyma-sparing surgical treatment (endocystectomy) are still under debate. This is no surprise, considering the high variability of CE presentations. Development of these criteria requires well-described standardized techniques for the principle surgical approaches. For our highly standardized endocystectomy protocol, we selected well-defined, uncomplicated, staged cysts (WHO cyst stages CE1, 2, 3a, 3b) to minimize heterogeneity and interacting factors. Judging the efficacy and complications of individual surgical treatment modalities has been difficult in published studies because of limited cyst staging, heterogeneous inclusion criteria and study endpoints, and short follow-up periods. A recent systematic review revealed that 71.2% of the papers published on hepatic CE did not mention any classification at all and WHO classification were only mentioned in 14% of papers [21]. Our endocystectomy protocol has been developed with a good understanding of the architecture of CE cysts and we have concentrated on CE-specific steps, most importantly the inactivation and spillage control of highly active cyst content, which determines recurrence. Identification and closure of biliary fistulas, management of biliomas and seromas etc. are not CE-specific but are widely encountered in liver surgery. Interpretation of published studies is complicated by the fact that patients in need of surgery are treated in a wide range of health systems in countries with varying resources and skills of surgeons, anesthetists, and other health-service staff. This means that non-CE-specific factors also play a role in managing patients. Therefore, highly standardized surgical protocols and standardization of the practice are of high importance for improving the surgical outcomes of hepatic CE worldwide, and for determination of criteria for assigning patients to different surgical techniques.

With these limitations in mind, the postoperative morbidity and mortality rates in our endocystectomy patient series are similar to those after radical surgery reported in the meta-analysis of He et al., (19.0% vs 17.7% and 0% vs 0.8%, respectively) [22]. The parenchyma-sparing endocystectomy is more feasible than resection in a wide range of general surgical units with minimal operative trauma. However, 20% sodium chloride is used during conservative surgical procedures, which increases the risk of chemical cholangitis if not used with greatest care.

In our series, surgical complications occurred in 14.2% of patients. The median number of cysts per patient was two (range: 1–8) and 47 endocystectomies were performed in total. The risk of surgical complications depended on the number of cysts per patient and increased with the number of endocystectomies performed per patient. Therefore, reporting the rate of surgical complications by total number of cysts operated on appears to be the more relevant figure. The total and surgical complication rates per number of endocystectomies performed were 8.5% and 6.4%, respectively. No CE recurrence was observed during the follow-up period, suggesting that this procedure is efficacious and safe from a surgical point of view. However, longer follow-ups are required to accurately determine the recurrence rates of hepatic CE stratified by the various conservative and radical surgical procedures.

Bile leakage is the most prevalent complication of endocystectomies. A meta-analysis identified no significant differences in bile leakage in patients treated with radical and conservative surgery. In contrast, individual reports have demonstrated a higher risk of bile leakage after conservative surgery. Wang et al. [19] suggested on the basis of a systematic review that bile leakage tests are useful and do not have adverse effects on the patients. The White test is strongly recommended to identify cysto-biliary communications and to ensure that all leaks are closed. In addition, over sewing the cyst capsule margin prevents bile leakage.

In summary, this highly standardized endocystectomy protocol (a) removes all biologically active CE cyst content from the residual cavity that would cause local recurrence, and (b) avoids intraabdominal and intravascular spillage of infectious CE material to prevent abdominal and systemic dissemination. Perioperative benzimidazole prophylaxis may be beneficial; however, the efficacy of this treatment in preventing recurrence in the event of spillage remains to be determined. In the experience of our center over the past 20 years, the close interdisciplinary collaboration between tropical medicine/infectious disease specialists, interventional radiologists, endoscopists, and surgeons has been key to the development of treatment strategies, refinement of intervention techniques, and adaptation of treatment modalities to the needs of individual patients with this highly complex disease.
